# ﻿*Oxytropis jamsranii* (Fabaceae, Papilionoideae, Astragaleae), a new species of section Xerobia from Mongolia

**DOI:** 10.3897/phytokeys.265.172785

**Published:** 2025-11-04

**Authors:** Shukherdorj Baasanmunkh, Dariganga Munkhtulga, Batlai Oyuntsetseg, Keum Seon Jeong, Hyeok Jae Choi

**Affiliations:** 1 Department of Biology and Microbiology, Changwon National University, Changwon 51140, Republic of Korea Changwon National University Changwon Republic of Korea; 2 Department of Biology, School of Arts and Sciences, National University of Mongolia, Ulaanbaatar 14201, Mongolia National University of Mongolia Ulaanbaatar Mongolia; 3 Sejong National Arboretum, Sejong 30106, South Korea Sejong National Arboretum Sejong Republic of Korea

**Keywords:** Flora of Mongolia, new species, Oxytropis, taxonomy, Xerobia

## Abstract

The genus *Oxytropis* L. (Fabaceae) comprises over 600 accepted species, ranking amongst the largest in the family. Nearly 100 *Oxytropis* taxa have been reported from Mongolia, spanning five subgenera and 20 sections in Mongolia. In this study, a new species, *Oxytropis jamsranii* Munkht. & Baasanm., from the Ikh Bogd Mountains in Mongolia, is described and illustrated. Based on morphological characteristics, it belongs to the section Xerobia. Although similar to *O. leptophylla* and *O. sobolevskajae* within the section, *O. jamsranii* is clearly distinguished by its indumentum, raceme structure, leaflet number and shape and corolla colour. The first occurrence of *O. sobolevskajae* — previously known only from the Republic of Tuva of Russia — in the flora of Mongolia is also reported here. Taxonomic nomenclature, morphological descriptions, distribution maps and field photographs for the new species and its two close relatives are provided.

## ﻿Introduction

The genus *Oxytropis* L. (locoweed), comprising more than 600 accepted species (Malyshev 2008; POWO 2025), is one of the largest and most diverse genera in Fabaceae (Malyshev 2008). Its native range spans the subarctic and temperate regions of the Northern Hemisphere (POWO 2025). In Mongolia, nearly 100 *Oxytropis* taxa have been reported, representing five subgenera and 20 sections (Ulziykhutag 2003; Malyshev 2008; Baasanmunkh et al. 2022). Recently, a new species *O. oyunmaae* Munkht. & Baasanm., was described from the Khusvgul Region of Mongolia (Baasanmunkh et al. 2025a).

The section Xerobia Bunge, comprising montane xerophytes, includes 27 species distributed across Central Asia and Asian Russia (Malyshev 2008). Amongst these, 15 species have been reported from Mongolia, including four endemics and eight subendemics (Baasanmunkh et al. 2021, 2022). This section is characterised by imparipinnate leaves with 2–8 pairs of leaflets and racemes bearing 1–8 flowers. The flowers are large (20–30 mm long), with sessile or stipitate (stipe to 5 mm) legumes that are ovoid or cylindrical, 5–27 mm and membranous to thickly leathery (Polozhii 1994; Malyshev 2008; Zhu et al. 2010). Recent studies have examined genetic diversity and phylogenetic relationships (Kholina et al. 2021) as well as natural selection on leaf shape (Wang et al. 2021) in several species of section Xerobia from China, Siberia and Mongolia. Within the section, only one species, *Oxytropis sobolevskajae* Pjak, has been described from the Republic of Tuva, Russia (Pyak 2014).

In the present study, a new species, *O. jamsranii*, from section Xerobia in Mongolia, is described, based on extensive morphological evidence. Additionally, *O. sobolevskajae* is newly recorded for the Mongolian flora.

## ﻿Materials and methods

Field surveys for collecting *Oxytropis* specimens and detailed field photographs have been conducted across Mongolia since 2020, yielding over 200 herbarium specimens deposited in the UBU (Herbarium of the National University of Mongolia). A targeted expedition was undertaken in the Ikh Bogd Mountains, Bayankhongor Province, from 2–14 July 2025; this southern Mongolian range reaches a maximum elevation of 3,600 m. Additional herbarium specimens were examined from the Herbaria ALTB, LE, MW, NS, GFW, UBA and UBU (Thiers 2023) and FloraGREIF (https://floragreif.uni-greifswald.de). Photo observations of selected *Oxytropis* species were cross-verified through the “Flora of Mongolia” project on iNaturalist platform (https://www.inaturalist.org/projects/flora-of-mongolia; Baasanmunkh et al. (2025b)). A distribution map was produced, based on these herbarium specimen records using ArcGIS (Esri 2012).

## ﻿Results and discussion

In this study, a new species, *Oxytropis jamsranii* Munkht. & Baasanm., is described from the Ikh Bogd Mountains, Jinst Soum, Bayankhongor Province, Mongolia. In addition, *O. sobolevskajae* is reported for the first time from the Mongolian flora. Taxonomic notes for three species in section Xerobia including *O. jamsranii*, *O. leptophylla* (Pall.) DC. and *O. sobolevskajae* are given.

### ﻿Taxonomic treatment

**Oxytopis subgenus Oxytropis , section Xerobia Bunge, subsection Ampulla**

#### ﻿ Oxytropis jamsranii

Taxon classificationPlantaeFabalesFabaceae

Munkht. & Baasanm. sp. nov.

702F7ED0-2AFA-5B08-B3A0-23DAF77E4922

urn:lsid:ipni.org:names:77371484-1

Fig. 1

##### Diagnosis.

*Oxytropis jamsranii* is morphologically most similar to *O. leptophylla* and *O. sobolevskajae* within section Xerobia, but differs in having 3-foliolate leaves with lanceolate, short-cuspidate leaflets 10–15 mm long [vs. *O. leptophylla*, 4–6 pairs, linear, obtuse or acute, 5–10 (20) mm; vs. *O. sobolevskajae*, 2–3 pairs, linear or linear-oblong, slightly acute, 10–35 mm long]; calyx 12–14 mm long, with dense, long white trichomes (vs. *O. leptophylla*, 8–11 mm long, with soft distant white, with some black hairs; vs. *O. sobolevskajae*, 11–13 mm long, with black, appressed, sinuous hairs and more sparse, long, white, squarrose, slightly curved hairs) (Table 1).

**Figure 1.**
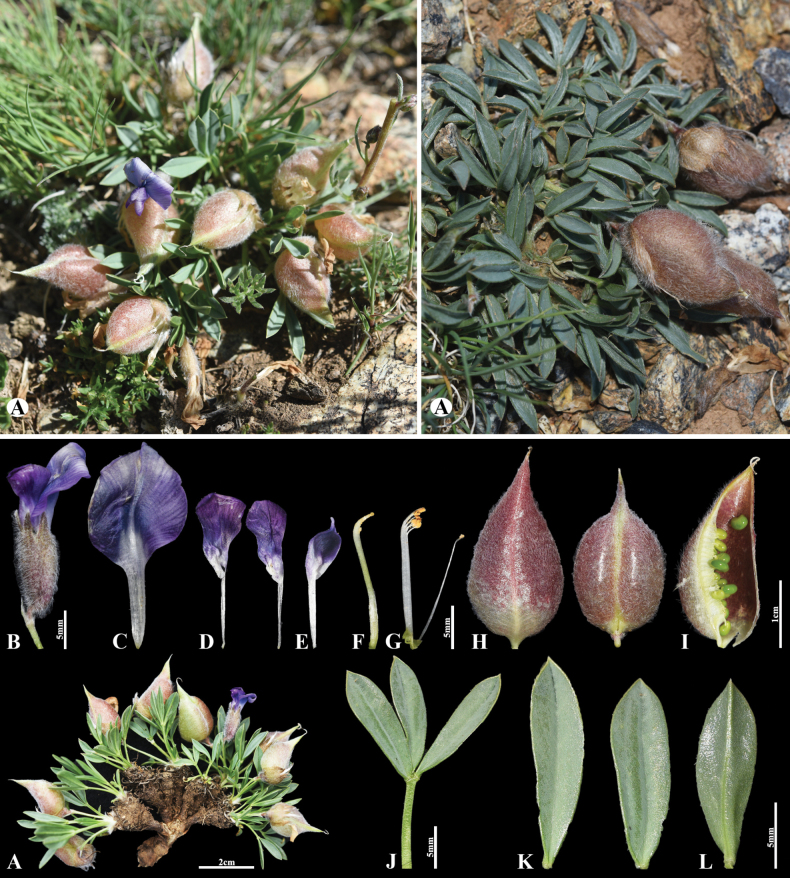
*Oxytropis jamsranii* Munkht. & Baasanm., sp. nov. A. General habits; B. Flower; C. Standard; D. Wings; E. Keel; F. Pistil; G. Stamens; H. Pods; I. Pod valve; J. Leaves; K. Leaf, adaxial view; L. Leaf, abaxial view (photo credits: D. Munkhtulga).

**Table 1.** Morphological comparisons of three Oxytropis species in section Xerobia in Mongolia.

Characteristics	O. jamsranii	O. leptophylla	O. sobolevskajae
Plants	subglabrous	almost glabrous	white pubescence
Raceme	1–2-flowered	2–5-flowered	1–2-flowered
Leaves	2–3 cm long, glabrous or diffuse sparsely-haired; leaflets 3	1–2 cm or 7–10 cm long, glabrous; leaflets 4–6 pairs	1.5–6.5 cm long, densely covered with appressed, slightly squarrose hairs; leaflets 2–3-paired
Corolla	purple-bluish-purple	raspberry-red pink	whitish-pink or light crimson
Pods	broad-ovate, 20–30 mm long	broad-ovate, 15–25 mm long	globose-ovate, 17–25 mm long

##### Type.

**Figure 2.**
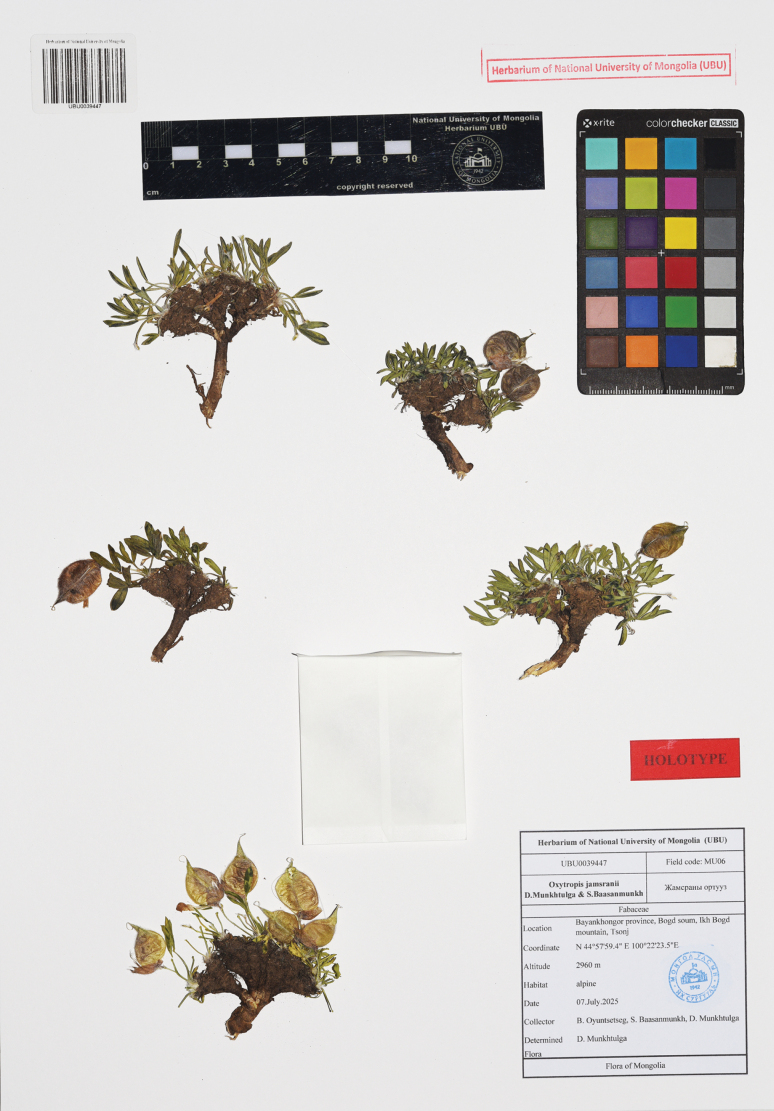
Holotype of *Oxytropis jamsranii* Munkht. & Baasanm (UBU0039447).

**Mongolia** • Bayankhongor Province, Bogd Soum, 44°57'59.4"N, 100°22'23.5"E, 2,960 m a.s.l., 07 July 2025, *B. Oyuntsetseg*, *S. Baasanmunkh & D. Munkhtulga MU06* [Holotype UBU0039447! (Fig. 2)].

##### Description.

Acaulous perennial herb, ca. 5 cm tall, forming small mats; with very short caudex branches; herbage grey-green, subglabrous. Stipules scarious, appressed-pilose, ciliate along margins. Leaves 2–3 cm long, slightly shorter than or equalling peduncles, petiole and rachis glabrous or sparsely appressed white long trichomes. Leaflets 3, lanceolate, short-cuspidate, 10–15 × 3–4 mm, glabrous, rarely appressed white-pilose on both surfaces. Racemes 1- or 2-flowered; peduncles appressed sparsely white-haired. Bracts linear, 2–3 × ca. 1.5 mm, greenish, leaf-like, densely sericeous-pubescent. Calyx membranous, grey-green to dark purple, tubular, 12–14 mm long, densely covered with long white trichomes; lobes acuminate, 2.5–3 mm. Corolla purple to bluish-purple; standard 20–22 mm long with broad-oval, undivided lamina; wings 16–18 mm long, shorter than standard, apex obliquely emarginated; keel 13–15 mm, with a beak ca. 2 mm at apex. Legume membranous, broadly ovate, inflated and cyst-like, 1-locular, 20–30 (including a 3–5 mm long beak) × 12–15 mm, with a ventral septum up to 2.5 mm broad, covered with white, squarrose, soft hairs.

##### Distribution.

Endemic to Mongolia (Fig. 5).

##### Habitat.

This species occurs in gravelly alpine areas and montane grasslands.

##### Etymology.

The species is named in honour of Prof. Tseden Jamsran (Цэдэн Жамсран, born in 1935), a distinguished Mongolian botanist who made significant contributions to the study and documenation of the flora of Mongolia (such as Flora of the Gurvan Saikhan Mountains).

#### ﻿ Oxytropis leptophylla

Taxon classificationPlantaeFabalesFabaceae

(Pall.) DC., Astragalogia 77 (ed. quarto), no. 12 (1802)

3F61B928-3424-5FC8-B5B4-B61DC8B79FB1

Fig. 3

##### Diagnosis.

Morphologically, *O. leptophylla* is most similar to *O. diversifolia* E.Peter. but differs in having leaves 2–10 cm long, 9–13-foliolate (vs. leaves 3–5 cm long, 3-foliolate), racemes compact to rather lax, 2–5-flowered (vs. racemes 1–2-flowered).

**Figure 3.**
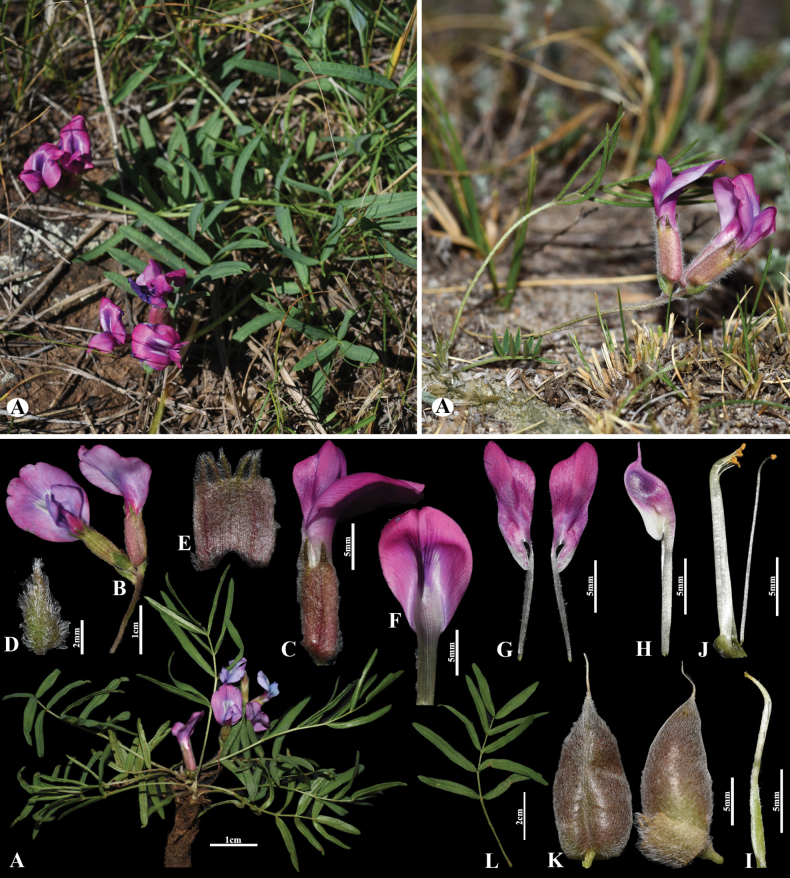
*Oxytropis leptophylla* in Mongolia. A. General habits; B. Raceme; C. Flower; D. Bract; E. Calyx; F. Standard; G. Wings; H. Keel; I. Pistil; J. Stamens; K. Pods; L. Leaves (photo credits: D. Munkhtulga).

##### Type.

**Russia** • Eastern Siberia, Buryatia: “≡ *Astragalus leptophyllus* Pall. Itin. III. s. l., s.d. fl., fr., Herb. Pallas” [Lectotype BM000958890, designated by Byalt and Sytin (2022); Syntype M0185403].

##### Distribution.

China (north-central), Mongolia (east) and Russia (Krasnoyarsk, Chita, Tuva) (POWO 2025).

##### Habitat.

This species occurs in sandy steppes and rocky steppe slopes.

##### Additional specimens examined.

Mongolia • East Mongolia Region: Sukhbaatar Province, Erdenetsagaan Soum, Zotol-Khan Mountain, 14 June 1980, *I. A. Gubanov 5669* (MW0183848, MW0183848); • Khentii Province, Bayankhutag Soum, Zaan-Shiree Mountain, 47°14'43.9"N, 111°33'59.0"E, 1,200 m a.s.l., 19 June 1987 *V. I. Grubov*, *E. Ganbold*, *A. L. Budantsev*, *R. V. Kamelin & Sh. Dariimaa 17* (MW0183847); • Dornod Province, Khalkhgol Soum, Numrug River, 47°05'00.0"N, 119°30'00.0"E, 800 m a.s.l., 29 June 1987, *V. I. Grubov*, *E. Ganbold*, *A. L. Budantsev*, *R. V. Kamelin & Sh. Dariimaa 746* (MW0183845); • Dornod Province, Khalkhgol Soum, Numrug River, 46°59'18.0"N, 119°23'34.2"E, 877 m a.s.l., 17 July 2017, *B. Oyuntsetseg & S. Baasanmunkh s.n.* (UBU0011943); • Sukhbaatar Province, Erdenetsagaan Soum, Bichigt Boomt, 45°43'01.9"N, 116°11'15.5"E, 912 m a.s.l., 08 August 2023, *B. Oyuntsetseg*, *D. Munkhtulga & A. Undruul EP13* (UBU0035024); • Dornod Province, Khalkhgol Soum, Sumiin Khooloi, 47°15'1852.04"N, 118°59'32.59"E, 910 m a.s.l., 20 May 2024, *D. Munkhtulga s.n.* (UBU0040642, UBU0041010).

#### ﻿ Oxytropis sobolevskajae

Taxon classificationPlantaeFabalesFabaceae

Pjak, Nordic J. Bot. 32(2): 139 (2013)

722A0F8F-AD71-5977-BBCD-707A91688408

Fig. 4

##### Diagnosis.

Morphologically, *O. sobolevskajae* is most similar to *O. leptophylla*, from which it differs in several key characteristics: leaflets 3–10-paired, abaxially sparsely appressed-hairy, adaxially glabrous (vs. 2–3-paired, abaxially rather densely appressed-hairy, adaxially sparsely hairy or glabrous); and inflorescences equalling the leaves, with flowers in umbellate racemes of 2–5 (vs. clearly shorter than leaves, flowers solitary or rarely 2) (Pyak 2014). Additionally, *O. sobolevskajae* occurs only in western Mongolia, whereas *O. leptophylla* is found in central and eastern Mongolia. *O. sobolevskajae* is also similar to *O. klementzii* N.Ulziykh., but differs in its inflated, thinly membranaceous, globose-ovate pods with a long beak, covered with white, squarrose, slightly sinuous, soft hairs (vs. hard, thickly coriaceous, nutlet-shaped pods covered with pubescent tomentose).

**Figure 4.**
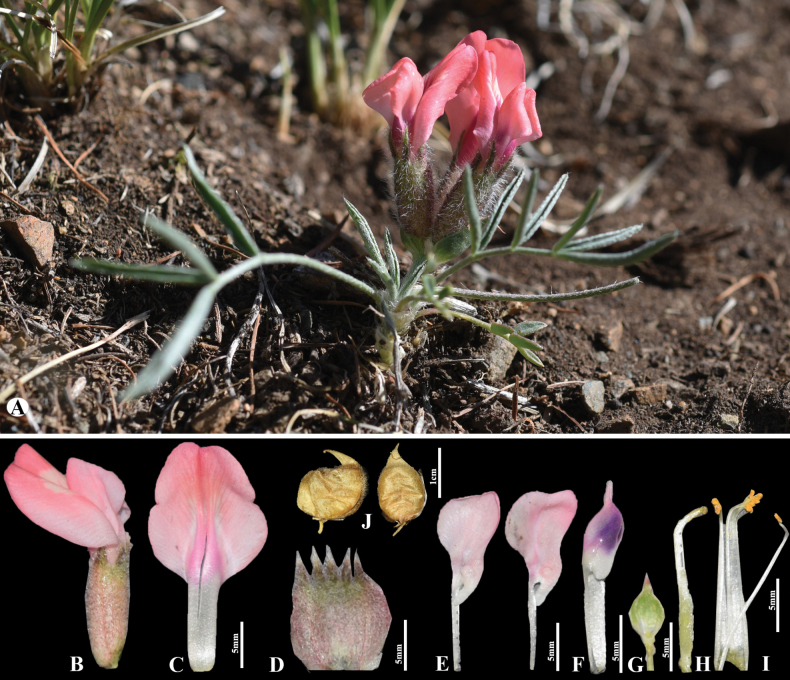
*Oxytropis sobolevskajae* in Mongolia. A. General habit; B. Flower; C. Standard; D. Calyx; E. Wings; F. Keel; G. Bract; H. Pistil; I. Stamens; J. Pods (photo credits: D. Munkhtulga).

##### Type.

**Russia** • Republic of Tuva: Pii-Khem District, Seserlig River Valley, near the bridge, north-exposed steppe slopes with sparse bushes, 51°52'12"N, 94°21'16"E, 759 m a.s.l., 1 Jun 2009, *A. I. Pyak s.n.* (Holotype: TK, Isotypes: LE, NS).

##### Distribution.

According to the distribution map provided by Pyak (2014), the Tuva population of this species occurs near the border with western Mongolia. Field expeditions were conducted in western Mongolia adjacent to the Tuva border, where we newly found *O. sobolevskajae* in Malchin Soum, Uvs Province.

**Figure 5.**
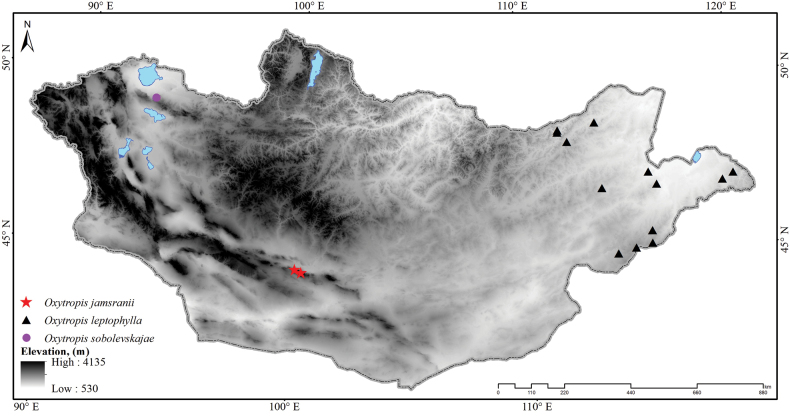
Distribution map of three *Oxytropis* species in Mongolia. *Oxytropis jamsranii* (red stars), *O. leptophylla* (black triangle), *O. sobolevskajae* (pink circle).

##### Habitat.

This species occurs in petrophytic meadows and rocky steppe areas.

##### Additional specimens examined (New record).

• Uvs Province, Malchin Soum, 49°41'17.9"N, 93°15'39.4"E, 1,622 m a.s.l., 31 May 2024, *B. Oyuntsetseg*, *G. Bayarmaa & D. Munkhtulga West2409* (UBU0036128).

## Supplementary Material

XML Treatment for Oxytropis jamsranii

XML Treatment for Oxytropis leptophylla

XML Treatment for Oxytropis sobolevskajae

## ﻿Additional information

### Use of AI

No use of AI was reported.

### Author ORCIDs

Shukherdorj Baasanmunkh https://orcid.org/0000-0003-4224-9376

Dariganga Munkhtulga https://orcid.org/0009-0009-0175-203X

Batlai Oyuntsetseg https://orcid.org/0000-0003-3772-3301

Keum Seon Jeong https://orcid.org/0009-0008-8577-9493

Hyeok Jae Choi https://orcid.org/0000-0001-6315-0071

### Data availability

All of the data that support the findings of this study are available in the main text.
